# The relation between language and arithmetic in bilinguals: insights from different stages of language acquisition

**DOI:** 10.3389/fpsyg.2015.00265

**Published:** 2015-03-13

**Authors:** Amandine Van Rinsveld, Martin Brunner, Karin Landerl, Christine Schiltz, Sonja Ugen

**Affiliations:** ^1^Education, Culture, Cognition and Society, Institute of Cognitive Science and Assessment, University of LuxembourgWalferdange, Luxembourg; ^2^Berlin-Brandenburg Institute for School Quality, Free University of BerlinBerlin, Germany; ^3^Department of Psychology, University of GrazGraz, Austria; ^4^Luxembourg Center for Educational Testing, University of LuxembourgLuxembourg, Luxembourg

**Keywords:** numbers, language learning, bilingualism, arithmetic, addition

## Abstract

Solving arithmetic problems is a cognitive task that heavily relies on language processing. One might thus wonder whether this language-reliance leads to qualitative differences (e.g., greater difficulties, error types, etc.) in arithmetic for bilingual individuals who frequently have to solve arithmetic problems in more than one language. The present study investigated how proficiency in two languages interacts with arithmetic problem solving throughout language acquisition in adolescents and young adults. Additionally, we examined whether the number word structure that is specific to a given language plays a role in number processing over and above bilingual proficiency. We addressed these issues in a German–French educational bilingual setting, where there is a progressive transition from German to French as teaching language. Importantly, German and French number naming structures differ clearly, as two-digit number names follow a unit-ten order in German, but a ten-unit order in French. We implemented a transversal developmental design in which bilingual pupils from grades 7, 8, 10, 11, and young adults were asked to solve simple and complex additions in both languages. The results confirmed that language proficiency is crucial especially for complex addition computation. Simple additions in contrast can be retrieved equally well in both languages after extended language practice. Additional analyses revealed that over and above language proficiency, language-specific number word structures (e.g., unit-ten vs. ten-unit) also induced significant modulations of bilinguals' arithmetic performances. Taken together, these findings support the view of a strong relation between language and arithmetic in bilinguals.

## Introduction

Although every human can manipulate approximate numerical quantities independently from language (Xu and Spelke, 2000), acquiring and mastering symbolic representations of exact quantities critically depends on language and instruction. Amazonian tribes who have restricted or no number words for quantities larger than five (or even two) impressively illustrate the importance of language for exact quantity representations. While their members can handle and manipulate large numerosities approximately, they are not able to process and represent them exactly (Gordon, 2004; Pica et al., 2004). Formal education enables the acquisition of exact number representations through labeling sets using distinct number names (Fuson et al., 1982). In other words, exact numerical quantities are learned through the use of language (Le Corre and Carey, 2007), and consequently exact number processing remains under the influence of language long after exact number representation acquisition. Recent studies demonstrated that basic processes such as number comparison are performed in slightly different ways depending on task language (Nuerk et al., 2005; Macizo et al., 2010; Van Rinsveld et al., 2012). Yet language plays an especially crucial role in more complex numerical computations, such as arithmetic problem solving. In the present study, we investigated whether and how the progressive acquisition of multiple languages modulates arithmetic problem solving in bilinguals.

### Language and arithmetic

Several studies provide strong evidence for an involvement of language in exact arithmetic (Spelke and Tsivkin, 2001). Exact calculations, contrary to approximate number processing, is thought to be represented in a specific language-coded format. Neuropsychological studies highlighted that the preservation of language is in fact necessary for arithmetic problem solving, as many authors reported an association between acalculia and aphasia (e.g., Delazer et al., 1999; Basso et al., 2000, 2005, but see Rossor et al., 1995; Cappelletti et al., 2001; Baldo and Dronkers, 2007). In the same way, neuro-imaging studies have shown that exact calculation tasks systematically activate specific language areas, arguing for an exact language-dependent system as opposed to a language-independent approximate system for number representations (Dehaene et al., 1999; Cohen et al., 2000; Stanescu-Cosson et al., 2000; Gruber et al., 2001; Venkatraman et al., 2006, but see Pesenti et al., 2000; Zago and Tzourio-Mazoyer, 2002; Benn et al., 2012).

Language is undoubtedly needed to build exact quantity representations, yet it still has to be clarified for what specific aspect of calculation language plays a crucial role. Heterogeneous solving strategies and processes can be involved in calculation depending on task difficulty (Beishuizen, 1993). Language may consequently affect distinct calculation types differentially. For that matter, it is important to separately examine the specific role played by language in each of the two classically distinguished arithmetic solving strategies. On the one hand, we distinguish simple calculations that are generally composed of one-digit operands (i.e., operands <10). For these problems it is widely accepted that learning and practice lead to a direct retrieval of their solutions from memory, as so-called “arithmetic facts” (Ashcraft, 1992; McCloskey, 1992; Fayol and Thevenot, 2012). However, there is less agreement concerning the storage format of these arithmetic facts: they could be represented in an abstract semantic format (McCloskey et al., 1985), a verbal format (Campbell, 1994; Dehaene and Cohen, 1995) or in a format depending on individual subject's preferences (Noel and Seron, 1993). Moreover, the importance of language in arithmetic fact retrieval is modulated by operation-type: simple addition and multiplication, in comparison to subtraction and division, especially rely on verbally coded facts probably because addition and multiplication facts are more often learned and used in their verbal code than subtraction and division (Lemer et al., 2003).

More complex calculations (i.e., operands >10), on the other hand, cannot directly be retrieved from memory but they require mental computations to be solved. These computations mainly rely on working memory resources to execute solving strategies, keep intermediate solutions in memory and update the final solution (Hitch, 1978; Ashcraft, 1995). According to Logie et al. (1994), the phonological loop of Bladdeley's working memory model (Baddeley, 1992) is used in mental calculation to verbally repeat the numbers. Studies using articulatory suppression during complex calculation have shown that phonological mediation occurs, especially when some elements of the problems disappear after a short presentation time (Fürst and Hitch, 2000). Moreover, a study with English-Welsh bilinguals found longer response times and more errors when calculations were performed in Welsh than in English, due to longer number words in the former (Ellis and Hennelly, 1980). Klessinger et al. (2012) revealed that the impact of number word lengths on exact additions was especially prominent in less proficient calculators. Similarly, a neuro-imaging study confirmed the crucial role of working memory in complex calculation and suggested that the working memory components engaged in the (visual or verbal) solving process may depend on individual solving strategies (Delazer et al., 2003). Taken together, these findings suggest that language is important for arithmetic problem solving at different levels because arithmetic facts are potentially represented or retrieved from memory in a verbal format and complex arithmetic solving processes rely at least partially on verbal working memory components.

Language is crucial for exact representation of large numerosities and for exact arithmetic problem solving, at least during the acquisition of these abilities. The different number naming systems used in different languages can modulate numerical performances during acquisition stages of numerical cognition but also in adults, who have long acquired these abilities (Campbell and Xue, 2001; Chen et al., 2009). Specifically, the order of tens and units in two-digit number words is a characteristic of number naming systems that can directly affect arithmetic performances. Brysbaert et al. (1998) showed that additions presented in the format “21 + 4” were solved faster by French-speaking participants (21 is pronounced twenty and one since French number words follow the ten-unit order), whereas the same additions presented in the format “4 + 21” were solved faster for Dutch-speaking participants (21 is pronounced one and twenty since Dutch number words following the unit-ten order). Moreover, Göbel et al. (2014) reported that German-speaking children had a larger carry effect in additions compared to Italian-speaking children. They explained this result by the greater similarity between the Arabic digit notation and the order of tens and units in Italian number words than in German number words. Indeed, Arabic digit notation follows the same order (from left to right) than the tens-units order of Italian number words (e.g., “24” = twenty-four) but they are inverted in comparison to the unit-ten order of German number words (e.g., “24” = four-and-twenty).

Another difference between number naming systems from different languages is the use of a base-20 structure instead of base-10 structure for two-digit number words (e.g., in French and in Basque). Seron and Fayol (1994) highlighted the specific difficulties encountered by French-speaking children for 70 and 90 number words following this base-20 structure used in France, in comparison to Belgian French-speaking children who use base-10 structure for 70 and 90. The former took longer and made more errors than the latter when writing down Arabic digits in a number dictation task. The base-20 system seems to have an impact not only during development but also later since a study by Colomé et al. (2010) showed that adult Basque speakers are influenced by the base-20 system of their language when they solve addition problems (see also Salillas and Carreiras, 2014). Taken together, the structure of the number words in the language in which numbers are acquired appears to affect arithmetic performances during childhood and some influence on arithmetic computation even persists in adulthood.

### Bilinguals and arithmetic

Given the critical role of language in arithmetic problem solving, how people using several languages (e.g., bilinguals) calculate is a particularly intriguing question. Many models of language processing in bilinguals support the idea that bilinguals' languages are active in parallel at any time, occasioning mutual interferences between languages (e.g., Kroll et al., 2014). It is generally assumed that interferences of the dominant language during the use of the non-dominant language are more consequential than interferences in the opposite direction, and the relative asymmetry in this mutual influence is a function of bilingual proficiency (Bialystok, 2009; Kroll et al., 2013). Indeed, higher proficiency level in a language lessons potential interferences of other languages on it. From the reports in the literature about bilingual's arithmetic problem solving it appears that several elements concerning the relative mastery of languages (i.e., language proficiency) as well as the structure of the number words in the involved languages directly modulate bilinguals' performances in arithmetic. Relevant data concerning these two aspects are highlighted below.

Early studies in bilingual speakers provided first indications that arithmetic skills is related to language proficiency. They indeed observed systematic advantages in response time and accuracy when bilinguals calculated in their first compared to their second (and less proficiently mastered) language (Marsh and Maki, 1976; McClain and Huang, 1982; Geary et al., 1993). Frenck-Mestre and Vaid (1993) tested addition fact-verification tasks in bilinguals with correct-outcome problems but also false-outcome problems that could be related or unrelated to multiplication facts (i.e., 2 + 3 = 6 was a false-outcome addition related to a multiplication fact). The authors observed associative confusion when problems were presented in bilinguals' first language and in Arabic digits but not in bilinguals' second language, so that they argued in favor of automatic arithmetical fact retrieval in the first but not the second language.

More recently, neuro-imaging studies on late Chinese-English bilinguals suggested that the verbal code of the first language is needed to retrieve arithmetic facts when the network of arithmetic facts in second language is not sufficiently developed. Wang et al. (2007) observed that performing complex calculations in first and second languages rely on a common activation network, but with higher activations during calculations in second language. This was interpreted as evidence for extra language processing needs in second language; potentially translation of input from second into first language (Lin et al., 2011). Taken together, these bilingual studies point toward an advantage for both retrieving arithmetic facts and computing complex arithmetic problems in the first language, i.e., the language in which most bilinguals learned to do arithmetic.

However, the bilinguals tested in the aforementioned studies were all late bilinguals or clearly unbalanced bilinguals, so the picture may be a bit different in more balanced bilinguals or bilinguals who did not acquire arithmetic in their first language. Indeed, one study reported that highly proficient bilinguals produced arithmetic facts equally well in their two languages (Campbell and Epp, 2004). Moreover, a study with Philipino-English bilinguals reported better arithmetic fact verification performances in English number word presentation, which was their second language but also the language in which they learned arithmetic at school and which they reported as their preferred language for doing arithmetic (Bernardo, 2001). Furthermore, Salillas and Wicha (2012) provided evidence for strong associative networks between terms and solutions for problems in the language in which participants learned arithmetic, which was not necessarily their first language. Participants seemed to maintain these early-established networks in adulthood, independently of language proficiency. These results were supported by a recent study where bilinguals showed switching costs when they had to retrieve arithmetic facts in their untrained- vs. trained-language (Saalbach et al., 2013). Hence, when bilinguals solve arithmetic problems, the language in which arithmetic was learned might be even more critical than the first language or the language in which they are currently most proficient.

In sum, bilinguals' arithmetic performances can be modulated by language proficiency levels, language of math acquisition and number word structure of the respective spoken languages. However, we are still lacking extensive studies, which investigate the relation between these different factors and arithmetic performances in bilingual participants. Such approaches are nevertheless necessary to understand in detail how language contributes to numerical computations. It is for instance currently unclear whether in highly proficient bilinguals performance levels in arithmetic become equivalent for their two languages or whether they maintain an advantage for retrieving and/or calculating in the language of arithmetic acquisition. It is also not known what increasing language proficiency implies for simple and complex calculations. Finally, it remains to be explored how language-related differences in number word structures affect arithmetic performance in bilinguals (e.g., German units-tens vs. the French tens-units; German base-10 vs. French base-20 for number words between 70 and 99).

### The present study

One of the major issues when studying bilinguals is that there often are as many different stories and profiles of languages acquisition as individuals. However, age of acquisition and proficiency levels of languages in bilinguals may drastically influence various ranges of cognitive processes (e.g., Altarriba and Basnight-Brown, 2007). In the present study we took advantage of the unique German-French bilingual school system of Luxembourg in order to address the aforementioned questions concerning to the relation between language and arithmetic by tracking the development of addition solving in bilingual adolescents and young adults at five different stages of bilingual proficiency. Bilingualism is a major attribute of the Luxembourgish educational system, as German and French are both teaching languages. In primary school teaching is held exclusively in German, but during secondary school, teaching language progressively switches to French, so that the pupils become highly proficient both in German and French through their education.

We composed four samples of German-French bilingual pupils at different levels of Luxembourgish secondary school (i.e., grades 7, 8, 10, and 11) and one sample of German-French bilingual young adults (who had also attended secondary school in Luxembourg). All participants thus mastered both German and French. Pupil participants from grades seven to 11 incrementally improved their mastery in German and French, with a relative emphasis on French as this language was becoming their predominant teaching language. The young adults achieved the level of excellence in both French and German. Altogether this yielded a design encompassing five distinct stages of German-French bilingualism.

For empirical research on the interplay of language and arithmetic the bilingual context in Luxembourg is characterized by a double advantage. (a) Firstly, all participants of a given age-class have a similar exposure to each of the two languages, as they are all first taught in German and then in French. This allows composing large samples of bilingual participants that are homogenous in terms of duration and amount of exposure to each language. Moreover, although bilingual, all participants acquired arithmetic in German. (b) Secondly, German-French bilinguals are particularly interesting because German and French languages use inverted number word structures. Two-digit number words follow the units-tens order in German (e.g., “four-and-twenty”) but the tens-units order in French (e.g., “twenty-four” like in English).

The experimental tasks consisted in addition problems that participants had to solve both in German and in French during two separate sessions. Additions were presented in two different formats (i.e., visual presentation of Arabic digits and auditory presentation of number words) and consisting of two difficulty levels (i.e., simple and complex additions). Throughout the entire experiment participants had to give their answers orally in the language of the session. Thus, task language permeated task instructions as well as presentation and solution of the additions in the auditory format, whereas only task instructions and solution production were imbued by task language in the visual format. Based on the literature reviewed above a series of predictions concerning the influence of language on addition solving in German-French bilinguals could be derived. Moreover, we also formulated detailed proposals on how these language effects might express at different stages of bilingual proficiency.

#### Effects of calculation complexity on bilinguals' arithmetic solving

In simple additions participants are thought to retrieve the solution from memory (Ashcraft, 1995). Previous studies with bilinguals have shown evidences for early-encoded arithmetic facts in one of the bilinguals' languages (e.g., Frenck-Mestre and Vaid, 1993; Spelke and Tsivkin, 2001; Wang et al., 2007) underlined by format-depending representations (Dehaene and Cohen, 1995). Nevertheless, other studies have highlighted evidences for transferable facts from one language to the other in very proficient bilinguals (e.g., Campbell and Epp, 2004) suggesting a possible representation of numbers independent from any format or language of encoding (McCloskey et al., 1985). Consequently, it can be expected that highly proficient bilinguals (i.e., adults and older adolescent participants of the present study) retrieve addition facts equally well in German and French. Indeed, these participants should be proficient enough in French and/or have been sufficiently exposed to numbers in French to be able to solve the simple additions similarly in French as in German.

Language-related performance differences ought to predominantly arise with complex additions. Compared to simple calculations, more complex arithmetic problems are thought to rely on computational procedures composed of multiple processing steps (e.g., Fayol and Thevenot, 2012), which can be modulated by language proficiency but also by specific number word structures. With respect to task presentation format, language effects should be larger for auditory than visual presentation formats because the operands have to been kept in memory in the former (LeFevre et al., 2001). In line with the prominent role of language, we expected that participants of all proficiency levels solve complex additions better and faster in German than in French. Indeed all participants had acquired German earlier than French and German was also their language of arithmetic acquisition (Bernardo, 2001; Salillas and Wicha, 2012). At the highest bilingual proficiency levels this benefit should be reduced, but we anticipated that it might never be resorbed completely if the early constellation of bilingual proficiency is critical.

### Effects of number word structures on bilinguals' arithmetic solving

Performance differences that arise when bilinguals solve additions might also be due to the specific number word structure of the respective languages. To gauge the impact of the different two-digit number-naming systems used in French vs. German on arithmetic performance in the five different bilingualism proficiency groups, we investigated two aspects of the number words.

Firstly, we explored whether the particular *base-20 number word structure* used in French (but not in German) for numbers from 70 to 99 might impact arithmetic performances differentially across age-groups. Indeed, the number words under 70 follow the classical base-10 structure in both task languages, while the number words over 70 follow the base-10 structure in German but not in French (where they follow the base-20 structure). We expected to find a general problem size effect in both languages because arithmetic problems with larger numbers are assumed to be more difficult to solve than arithmetic problems with smaller numbers (Groen and Parkman, 1972). But more interestingly, we also assumed that additions involving numbers over 70 would be specifically difficult in French because of the base-20 structure (Seron and Fayol, 1994). This specific difficulty should thus be especially pronounced at lower French proficiency levels.

Secondly, we aimed to understand whether and how the *order of tens and units in number words* (i.e., tens-units in French vs. units-tens in German) plays a role in bilinguals' addition performances. As Pixner et al. (2011) reported that the number naming system used in a two-digit number transcoding task modulated the type of errors, we analyzed which errors bilingual participants made on complex additions across the different presentation formats and languages. Given the contrasting positions of units and tens in German and French it is plausible that the same bilingual participant makes errors that predominantly pertain to distinct value positions depending on the language in which the calculation is performed.

Testing these predictions on our unique German-French bilingual sample will allow us to better understand the relation between language and arithmetic in bilinguals and how this relation evolves with increasing bilingual proficiency levels. To the best of our knowledge, there are currently no studies that systematically investigated how the influence of number word structure on arithmetical performance evolves as a function of language proficiency. Taken together these original data should also yield new insights into the role of language in arithmetic and number processing in general.

## Methods

### Participants

A total of 193 bilingual participants were recruited for the present study. The sample was composed of 36 pupils from grade 7 (21 females; mean age of 12.2 years; *SD* = 0.36 years), 33 pupils from grade 8 (13 females; mean age of 13.2 years; *SD* = 0.58 years), 35 pupils from grade 10 (15 females; mean age of 15.5 years; *SD* = 0.66 years), 41 pupils from grade 11 (19 females; mean age of 16.4 years; *SD* = 0.72 years) and 48 young adults (34 females; mean age of 22.4 years; *SD* = 2.67 years).

All participants thus spoke Luxemburgish (an official language of Luxembourg which developed from a dialectal variant of German) or German as native language and attended the Luxembourgish school system in the highest academic track, which prepares for attending college and university. Moreover, all study participants (including the adults) had attended Luxembourgish primary school that starts with German as teaching language. From second grade of primary school on, all participants learned French as a second language. Importantly, students in grades 7 and 8 were taught mathematics in French, whereas students in grades 10 and 11 were not only taught mathematics but also all of their other courses in French (except the German and English language courses). Over the school years, relative exposure and proficiency in French thus progressively increased and tended toward bilingualism with high proficiency levels in both German and French in the highest grades. Consequently, the adult group was composed of young adult participants who had become highly proficient German-French bilinguals through their education.

Native language(s), the number of years spent in Luxembourgish schools and linguistic background (under the form of self-assessment of language proficiency) were checked in a short questionnaire before starting the experiment in order to ensure that all participants also had similar exposures to languages in these respects. Adults received 20€ for their participation. Informed consent was obtained from all participants.

### Stimuli

Eighty-four two-operand addition problems were presented during the entire experiment. The set was composed of 28 one-digit simple additions (e.g., 4 + 2) and 56 two-digit complex additions (e.g., 56 + 32). This stimulus set was split in four blocks of additions to be allocated to both presentation formats of the problems and to both language sessions: visual and auditory presentation of the numbers in the German session and visual and auditory presentation of the numbers in the French session.

Simple additions were composed of two one-digit operands ranging from 2 to 9. We excluded +1 additions and additions between the same operands (e.g., 7 + 7), resulting in a range of solutions from 5 to 17. The simple additions with carry (additions with a solution of 10 or more) and without carry (additions with a solution below 10) were equally distributed across the four blocks of additions.

Complex additions were composed of two two-digit operands ranging from 12 to 86 in order to keep solutions below 100. We excluded all additions including a zero or ties. Furthermore, problems with a repetition of the same digit between the operands or between one of the operands and the solution were excluded, resulting in a range of solutions from 35 to 98. The requirement of a carry to be solved (with or without carry), the position of the larger operand (left vs. right in visual presentation; first vs. second in auditory presentation) and the problem size (small when the solution ranged between 30 and 69 or large when the solution ranged between 70 and 98) were taken into account in the repartition of complex additions in the four blocks. Indeed, each block contained seven problems with carry and seven problems without carry, and seven problems of small size and seven problems of large size. In other words, among the small problems, half of them contained a carry and half of them did not, and the same for the large problems, so that problems with and without carry were distributed equally among problems of different sizes within each block. The assignation of the blocks to a presentation format and a language was balanced through participants. For instance, block 1 was be assigned to visual presentation of the French session for the first eight participants but the same block 1 was assigned to visual presentation of the German session for the next eight participants.

### Procedure

We ran the experiment on an Apple 13′ Macbook using Psyscope X B57 (Cohen et al., 1993) where voice onset times of responses were recorded with a voice key on the Iolab USB Button Box. As the voice key only recorded the response onset, the experimenter wrote the solutions down and pressed a key to start the next trial, which started after an inter-trial interval of 500 ms. The onset of the response time (RT)—measurement started when the stimulus presentation was completed.

In the visual presentation format, additions appeared on a white screen in black (Arial, font size 90) until participants responded. In the auditory presentation format, participants had to listen to the additions via headphones (in both ears). The length of auditory presentation was controlled between languages separately for simple and complex additions, so that the mean duration of auditory presentation did not differ between languages (see Table 1). In both presentation formats, participants had to respond orally by giving the solution in the microphone in the language of the task. This means that for auditory presentation of the additions, RT-measurement started at the offset of the second operand.

**Table 1:** **Mean duration of presentation of auditory additions in ms with standard deviation for each complexity level of the additions as a function of language**.

	**Simple additions**		**Complex additions**	
	***M***	***(SD)***	***M***	***(SD)***
German	1736	(80.1)	2582	(134.6)
French	1745	(169.8)	2574	(235.3)
Total	1740	(131.6)	2576	(191.8)

*Mean presentation time differences between languages were not significant neither for simple additions, t_*(54)*_ = 0.239; p = 0.812, not for complex additions, t_*(108)*_ = 0.148; p = 0.883*.

The testing was organized in two language sessions: participants performed both presentation formats first in one task language and then in the other. Order of presentation formats and task languages were counterbalanced between participants. Instructions and interaction with the experimenter remained in German or in French, according to the session. Participants were tested individually and were instructed to respond as accurately and as fast as possible. Seven training items preceded the 21 additions of each block. The entire experiment lasted about 50 min.

### Data processing

#### Effects of calculation complexity (simple vs. complex additions)

In order to track the development of arithmetic problem solving in bilingual children and adults, correct response times (RTs) and correct response rates (CRs) during experimental tasks were collected at five different stages of language proficiency. Training items were not included in the dataset, and we also excluded RTs of all trials below or above three standard deviations from the mean of each participant and from the group mean. We excluded 4% of the trials in this way before analyzing the RTs.

We ran a preliminary analysis of variance (ANOVA) on the RTs and the CRs including all additions participants had to solve with Complexity_2_ × Format_2_ × Task language_2_ as within-subject factors and Age-group_5_ as between-subject factor. The two levels of complexity were the simple one-digit operand vs. the complex two-digit operand addition problems; format referred to visual or auditory presentation of the additions; and task language was German or French (for instructions, presentation of the additions in the auditory format, and production of the answer). The age-group factor had the following levels: seventh graders, eighth graders, tenth graders, eleventh graders or young adults. The aim of this preliminary ANOVA was to see whether it was relevant to analyze both complexity levels (simple vs. complex addition) separately. Therefore, we only report results from the effect and interactions with the complexity factor. Then, we ran analyses of variance (ANOVA) on the RTs and the CRs separately for each type of additions: i.e., the simple one-digit additions and the complex two-digit additions. Within each ANOVA we used Format_2_ × Task language_2_ as within-subject factors and Age-group_5_ as between-subject factor.

#### Effects of number word structure

To investigate how arithmetic performance is influenced by the different structures of number words in German and French we conducted two additional analyses. Firstly we tracked the impact of the particular base-20 number word structure used in French but not in German for numbers from 70 to 99 on the arithmetic performances across age-groups. Therefore, we introduced one more factor in the ANOVA on complex additions: the problem size. We categorized the items in two levels of problem size according to whether problems involved or not a number over 70. Indeed the number words under 70 follow the classical base-10 structure in both task languages, whereas the number words over 70 follow the base-10 structure in German but the base-20 structure French. We thus ran an ANOVA with Problem size_2_ × Format_2_ × Task language_2_ as within-subject factors and with Age-group_5_ as between-subject factor.

Secondly, we focused on the impact of the order of tens and units (i.e., ten-unit in French vs. unit-ten in German) in two-digit number words on arithmetic performances. We analyzed the type of errors participants made across different presentation formats and languages when solving complex additions involving on two-digit numbers. Within each task language and format, we listed the rate of errors (%) for which only the ten-digit was false (“ten-error,” i.e., 34 instead of 24) and inversely, the rate of errors for which only the unit-digit was false (“unit-error,” i.e., 34 instead of 35). Other types of errors were not included in the analyses because we found less than 2% of each type. We ran an ANOVA on these error rates with Error type_2_ × Format_2_ × Task language_2_ as within-subject factors and with Age-group_5_ as between-subject factor. The two levels of the error type factor corresponded to “ten-error” and “unit-error” and the level of the other factors were the same as in the previous analyses.

## Results

### Effects of calculation complexity (simple vs. complex additions)

Preliminary ANOVA showed a strong effect of complexity on both RTs [*F*_(1, 184)_ = 893.961; *p* < 0.001; η^2^ = 0.829] and CRs [*F*_(1, 185)_ = 510.891; *p* < 0.001; η^2^ = 0.734]. Both in RTs and CRs, complexity modulated effects of language [RTs: *F*_(1, 184)_ = 177.873; *p* < 0.001; η^2^ = 0.492, CRs: *F*_(1, 185)_ = 64.294; *p* < 0.001; η^2^ = 0.258], format [RTs: *F*_(1, 184)_ = 43.034; *p* < 0.001; η^2^ = 0.190, CRs: *F*_(1, 185)_ = 235.634; *p* < 0.001; η^2^ = 0.560] and age-group [RTs: *F*_(4, 184)_ = 12.740; *p* < 0.001; η^2^ = 0.217, CRs: *F*_(1, 185)_ = 3.880; *p* = 0.005; η^2^ = 0.077]. We also observed a triple interaction between complexity, language and format [RTs: *F*_(1, 184)_ = 13.119; *p* < 0.001; η^2^ = 0.067, CRs: *F*_(1, 185)_ = 9.768; *p* = 0.002; η^2^ = 0.050]. Only in RTs, there was also a significant triple interaction between complexity, language and age-group [RTs: *F*_(4, 184)_ = 4.610; *p* = 0.001; η^2^ = 0.091]. Since all factors of the preliminary ANOVA interacted with complexity, we will directly report below separate analyses and results for both complexity levels.

#### Simple additions

**Figure 1:**
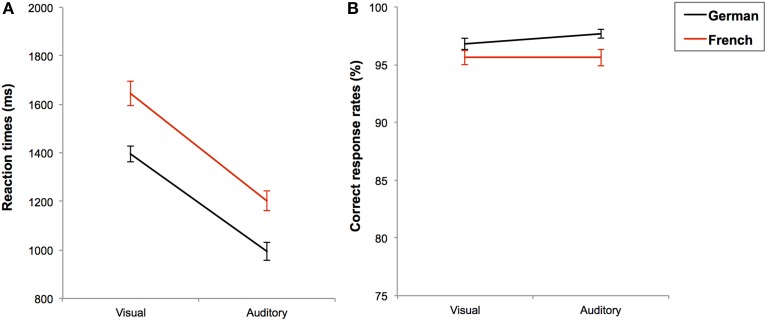
**Mean reaction times in ms (A) and mean correct response rates in percentages (B) with standard errors for the simple additions in each task language (black line for German and red line for French) as a function of presentation format**.

For the simple additions, overall mean RT was 1309 ms (*SE* = 32 ms) and overall mean CR was 96.4% (*SE* = 0.3%). We found an age-group effect on RTs [*F*_(4, 184)_ = 12.710; *p* < 0.001; η^2^ = 0.216] and on CRs [*F*_(4, 185)_ = 3.038; *p* = 0.019; η^2^ = 0.062], as participants solved the simple additions faster and more accurately with increasing age-group (see Table 2). Furthermore, simple additions were performed faster when they were presented in auditory than in visual format, *F*_(1, 184)_ = 171.992; *p* < 0.001; η^2^ = 0.483, but no difference between formats was observed in terms of CRs, *F*_(1, 185)_ = 0.737; *p* = 0.392; η^2^ = 0.004 (see Figures 1A,B). Thus, simple auditory additions were solved faster than visually presented ones, but correct response rates were similar for both formats.

**Table 2:** **Means of reaction times (RT) in ms and correct response rates (CR) in % with standard errors for each complexity level of the additions (simple vs. complex) and the general mean performances as a function of age-group**.

**Group**	**Simple additions**		**Complex additions**		**Total**	
	***M***	***(SE)***	***M***	***(SE)***	***M***	***(SE)***
**RT**						
Seventh graders	1638	(74.5)	5774	(285.9)	3706	(173.7)
Eighth graders	1408	(77.9)	4533	(298.9)	2970	(181.6)
Tenth graders	1358	(75.6)	4377	(290.0)	2867	(176.2)
Eleventh graders	1145	(69.7)	3691	(264.1)	2383	(162.5)
Adults	994	(63.6)	3098	(244.1)	2046	(148.3)
Total	1309	(32.4)	4294	(124.0)	2794	(75.5)
**CR**						
Seventh graders	94.8	(0.7)	74.5	(1.9)	84.7	(1.1)
Eighth graders	95.7	(0.8)	77.0	(1.9)	86.4	(1.2)
Tenth graders	96.9	(0.7)	77.7	(1.9)	87.3	(1.1)
Eleventh graders	96.5	(0.7)	82.1	(1.7)	89.3	(0.1)
Adults	98.0	(0.6)	84.9	(1.6)	91.5	(0.9)
Total	96.4	(.03)	79.2	(0.8)	87.8	(0.5)

**Figure 2:**
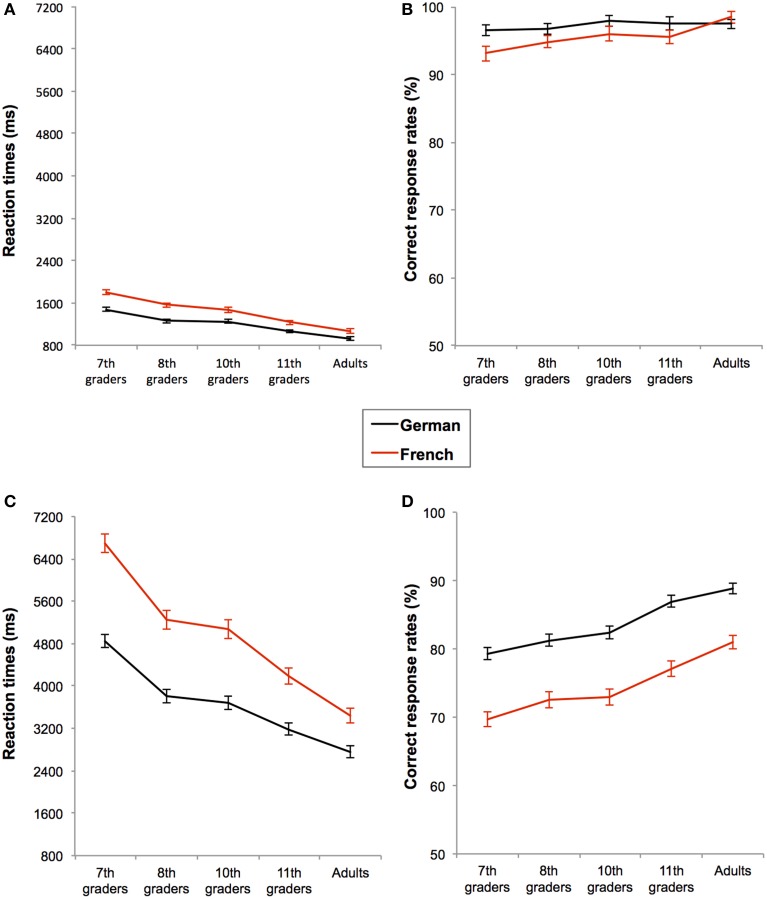
**Mean performances for simple additions (A, B) and for complex additions (C, D) in each task language (black line for German and red line for French) as a function of age-group**. Performances are measured in reaction times **(A, C)** and in correct response rates **(B, D)**. Bars represent standard errors.

Moreover, simple additions were performed faster in German than in French [RT: *F*_(1, 184)_ = 77.199; *p* < 0.001; η^2^ = 0.296], see Figure 2A. Participants also made fewer errors in German than in French [*F*_(1, 185)_ = 9.782; *p* = 0.002; η^2^ = 0.050], but this language effect on CRs was marginally modulated by the age-group (language × age-group: *F*_(4, 185)_ = 2.234; *p* = 0.067; η^2^ = 0.046), see Figure 2B. We decomposed this interaction by separately running a Format_2_ × Task language_2_ ANOVA on CRs in each age-group. It appeared that only the seventh graders were less accurate in French than in German, *F*_(1, 34)_ = 4.074; *p* = 0.050; η^2^ = 0.092, while all other age-groups performed with equal accuracy in both languages (all *F*'s < 1 and *p*'s > 0.05), see Figure 2B. No other interaction reached significance (all *F*'s < 1 and *p*'s > 0.05). In sum, participants solved simple additions faster in German than in French, but in both languages they performed the task faster when additions were presented in auditory than in visual format. In terms of accuracy, additions presented in both languages and presentation formats were performed equally well, except that seventh graders were less accurate in French than in German.

#### Complex additions

For the complex additions, overall mean RT was 4294 ms (*SE* = 124 ms) and overall mean CR was 79.2% (*SE* = 0.8%). We found an age-group effect on RTs [*F*_(4, 185)_ = 14.008; *p* < 0.001; η^2^ = 0.232] and on CRs [*F*_(4, 185)_ = 5.976; *p* < 0.001; η^2^ = 0.114], as participants from the older age-groups solved the complex additions faster and more accurately (see Table 2).

Regardless of task language complex additions were performed faster [*F*_(1, 185)_ = 4.997; *p* = 0.027; η^2^ = 0.026], and more accurately [*F*_(1, 185)_ = 245.736; *p* < 0.001; η^2^ = 0.571], when presented visually than in auditory format, see Table 3. Moreover, for CRs the format effect was modulated by age-group [format × age-group interaction: *F*_(4, 185)_ = 8.365; *p* < 0.001; η^2^ = 0.153]. Decomposition of this interaction showed that participants became more accurate with age for auditory presented additions. However, CRs remained similar across age-group for visually presented additions. This led to a progressively smaller error rate difference between visual and auditory formats with age-group (see Table 3).

**Table 3:** **Means of reaction times (RT) in ms and correct response rates (CR) in % with standard errors for each presentation format of the simple and complex additions as a function of age-group**.

**Group**	**Simple additions**				**Complex additions**			
	**Visual**		**Auditory**		**Visual**		**Auditory**	
	***M***	***(SE)***	***M***	***(SE)***	***M***	***(SE)***	***M***	***(SE)***
**RT**								
Seventh graders	1863	(86.9)	1414	(79.4)	5430	(260.2)	6118	(349.1)
Eighth graders	1577	(90.9)	1239	(83.0)	4485	(272.1)	4580	(365.1)
Tenth graders	1575	(88.2)	1142	(80.6)	4283	(263.9)	4470	(354.2)
Eleventh graders	1357	(81.3)	933	(74.3)	3682	(240.4)	3699	(322.6)
Adults	1231	(74.2)	757	(67.8)	3038	(222.1)	3158	(298.1)
Total	1520	(37.8)	1097	(34.5)	4183	(112.8)	4405	(151.5)
**CR**								
Seventh graders	94.6	(0.9)	95.1	(1.0)	86.2	(1.6)	62.8	(2.6)
Eighth graders	95.5	(0.9)	96.0	(1.0)	84.5	(1.6)	69.4	(2.7)
Tenth graders	96.6	(0.9)	97.3	(1.0)	85.2	(1.6)	70.2	(2.6)
Eleventh graders	96.3	(0.8)	96.7	(0.9)	87.3	(1.4)	76.8	(2.4)
Adults	97.9	(0.8)	98.1	(0.8)	89.1	(1.3)	80.8	(2.2)
Total	96.2	(0.4)	96.6	(0.4)	86.5	(0.7)	72.0	(1.1)

**Figure 3:**
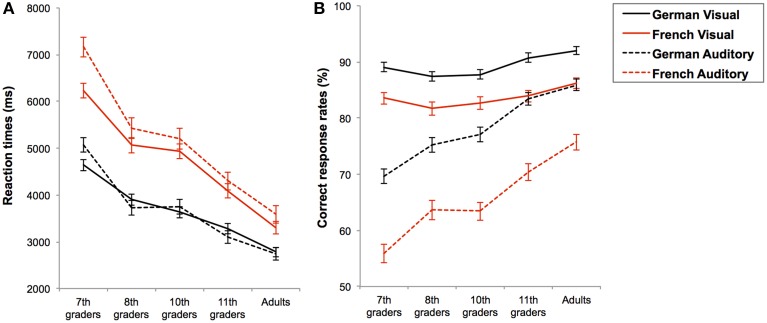
**Mean reaction times in ms (A) and mean correct response rates in percentages (B) with standard errors for complex additions in each task language (black line for German and red line for French) and in each presentation format (solid line for visual and dashed line for auditory) as a function of age-group**.

In general, complex additions were also performed faster and more accurately when the task language was German than when it was French, RT: *F*_(1, 185)_ = 201.922; *p* < 0.001; η^2^ = 0.522 and CR: *F*_(1, 185)_ = 113.630; *p* < 0.001; η^2^ = 0.381, see Figures 2C,D. However, this language effect was modulated by the presentation format, both in terms of RTs, *F*_(1, 185)_ = 10.729; *p* = 0.001; η^2^ = 0.055, and CRs, *F*_(1, 185)_ = 19.657; *p* < 0.001; η^2^ = 0.096. Firstly, results from the pairwise comparisons on the RTs showed that even if complex additions were always performed faster in German than in French, the effect of the format (i.e., visual vs. auditory) was only significant for French, *F*_(1, 185)_ = 9.867; *p* = 0.002; η^2^ = 0.051, but not for German, *F*_(1, 185)_ = 0.105; *p* = 0.746; η^2^ = 0.001. Hence, auditory-presented additions were performed slower than visually presented additions only in French, see Figure 3A. Secondly, results from the pairwise comparisons on the CRs showed that language-related accuracy differences were larger in auditory, *F*_(1, 185)_ = 109.919; *p* < 0.001; η^2^ = 0.373, than visual presentation format, *F*_(1, 185)_ = 28.536; *p* < 0.001; η^2^ = 0.134, see Figure 3B.

Thus, when task language was French, participants were slower for additions presented in auditory compared to visual format, but the presentation format did not modulate RTs in German. Additionally, additions of the German session were always solved more accurately than additions of the French session and this effect of task-language was more pronounced for additions presented in auditory format. Finally, regardless of presentation format, task language also interacted with the age-group on the RTs, *F*_(4, 187)_ = 5.317; *p* < 0.001; η^2^ = 0.103, but not on the CRs, *F*_(4, 187)_ = 0.194; *p* = 0.941; η^2^ = 0.004. Indeed, response times of both language sessions became increasingly similar with age, see Figure 2C.

When considering the above analyses it appears that the variability was different across age groups. Levene's test for homogeneity of variances across groups was indeed significant, as the younger groups performances were more heterogeneous than the older groups' (see standard errors reported in Table 2). This characteristic of the data is typical for transversal developmental comparisons, but it might have impacted the above-mentioned results and masked some interactions between age groups and task-language and/or presentation format effects. To cancel any potential influences of variance heterogeneity we therefore re-conducted the same analyses after a standardization of the data per age-group. The results of this additional analysis are detailed in the Annex 1 of Supplementary Material.

To sum up results on both raw and standardized data, bilingual participants of all five age groups solved *simple additions* faster in German than in French. Moreover auditory format simple additions were performed faster than visual format additions in both languages^1^. In contrast age group impacted the accuracy of simple addition solving, as seventh graders were overall less accurate in French than in German. This finding was confirmed by the z-score analyses, which revealed that only participants of the tenth grade onwards solved simple additions with equal accuracy in both languages, even if they remained always slightly faster in German than in French.

Furthermore, *complex additions* were performed faster and better in German than in French. Critically, age group impacted RT differences observed when bilingual participants solved complex additions in German compared to French. Nevertheless, additional results on z-scores showed that task-language effect on RTs did no longer interact with age-group after standardization of the data. Thus, the effect of task language on RTs and CRs remained constant proportionally across age-groups.

Concerning presentation format, even though the differences of RTs cannot be interpreted *per se*, results on CRs showed that participants made more errors in auditory format than in visual format, especially in French compared to German were the CRs difference between formats was smaller. And this effect interacted with age-group as participants became more accurate for auditory-presented additions with increasing age. This last interaction between format and age-group remained significant after standardization of the data, suggesting that participants' ability to solve auditory presented additions genuinely improves with age.

### Effects of number word structure

#### Base-10 vs. base-20 tens

Here we only report effects and interactions involving the problem size factor because other effects and interactions were already explained in detail in section Effects of Calculation Complexity (Simple vs. Complex Additions). In general, we observed lower CRs [*F*_(1, 185)_ = 85.196; *p* < 0.001; η^2^ = 0.315] and slower RTs [*F*_(1, 173)_ = 151.138; *p* < 0.001; η^2^ = 0.466] with problems over 70 than with problems under 70. Moreover, problem size interacted with the task-language both in RTs, *F*_(1, 173)_ = 16.327; *p* < 0.001; η^2^ = 0.086, and CRs, *F*_(1, 185)_ = 52.912; *p* < 0.001; η^2^ = 0.222.

**Figure 4:**
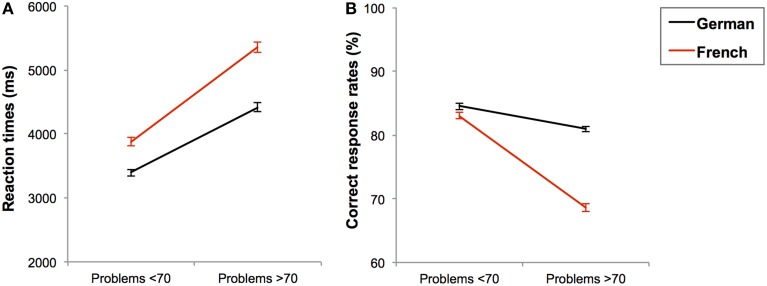
**Mean reaction times in ms (A) and mean correct response rates in percentages (B) with standard errors for complex additions solved in each task language (black line for German and red line for French) as a function of problem size (under and over 70)**.

To decompose this interaction, we ran pairwise comparisons. The problem size effect on the RTs was larger when the task language was French [*F*_(1, 173)_ = 91.565; *p* < 0.001; η^2^ = 0.346] than when it was German [*F*_(1, 173)_ = 64.836; *p* < 0.001; η^2^ = 0.273], see Figure 4A. In terms of CRs, problem size effect was only significant in French [*F*_(1, 185)_ = 105.613; *p* < 0.001; η^2^ = 0.363] but not in German [*F*_(1, 185)_ = 2.878; *p* = 0.091; η^2^ = 0.015], see Figure 4B. Further, the difference in CRs between German and French was smaller in problems under 70 [*F*_(1, 185)_ = 11.763; *p* = 0.001; η^2^ = 0.060] than in problems over 70 [*F*_(1, 185)_ = 140.200; *p* < 0.001; η^2^ = 0.431]. Finally, the problem size factor did not interact with any other factor, not even the age-group, all *F*s < 1 and *p*s > 0.1. Thus, task language strongly modulated the effect of problem size in the direction that problem size effects were more pronounced when the task was performed in French than in German.

When considering the above analyses it appears that participants generally responded slower in French, which was also their less mastered language. Thus, the greater problem size effect found in French could also be due to participants' weaker French proficiency, independently of the structure of number words in this language. To rule out this alternative explanation, we re-conducted this analysis after a standardization of the data per language, see results in Annex 2 of Supplementary Material. In summary, interactions of language and problem size remained significant after standardization of the data per language, suggesting that differences of problem size effect observed between languages in raw data are not a consequence of bilinguals' differences between languages in terms of language mastery.

#### Units-tens vs. tens-units

**Figure 5:**
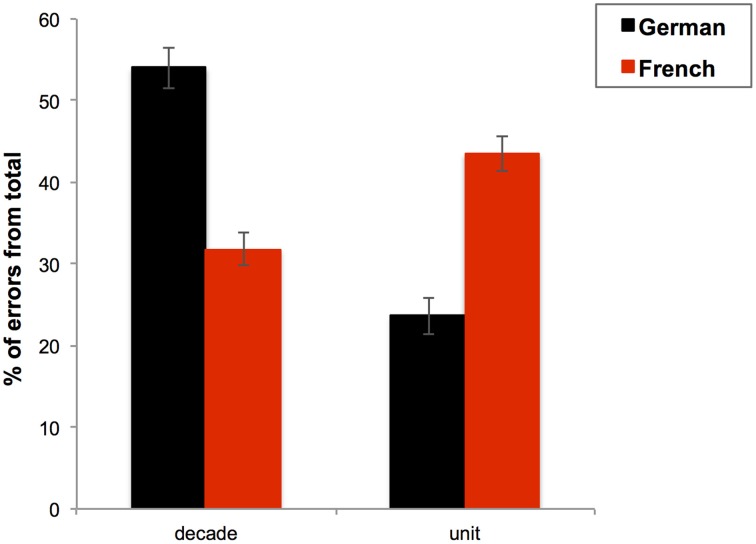
**Mean percentage of errors from the total errors (%) made on the decade digit or on the unit digit with standard errors for each task language (German in black and French in red)**. Bars represent standard errors.

In general, more errors were made on tens than units, *F*_(1, 110)_ = 10.283; *p* = 0.002; η^2^ = 0.085. Moreover, the task language × error type interaction was significant, *F*_(1, 110)_ = 56.194; *p* < 0.001; η^2^ = 0.338, and pairwise comparisons showed that there were more errors on the tens than on the units when additions were presented in German, *F*_(1, 110)_ = 50.108; *p* < 0.001; η^2^ = 0.313, but inversely, when additions were presented in French, there were more errors on the units than on the tens, *F*_(1, 110)_ = 9.594; *p* = 0.002; η^2^ = 0.080. Additionally, there were more errors on the tens in German than in French, *F*_(1, 110)_ = 48.293; *p* < 0.001; η^2^ = 0.305, and more errors on the units in French than in German, *F*_(1, 110)_ = 46.711; *p* < 0.001; η^2^ = 0.298, see Figure 5.

Furthermore, the format of presentation interacted with task language, *F*_(1, 110)_ = 7.783; *p* = 0.006; η^2^ = 0.066. Pairwise comparisons of German vs. French addition errors showed the same pattern of results in both presentation formation but more errors of both types were made in German than in French for the auditory presentation format, *F*_(1, 110)_ = 8.002; *p* = 0.006; η^2^ = 0.068. In contrast, more errors of both types were made in visual than in auditory presentation format in French, *F*_(1, 110)_ = 12.791; *p* = 0.001; η^2^ = 0.104. It should be noted that this last interaction does not change the conclusions yielded by the aforementioned complex additions results, as here the error rates only referred to percentage of errors on unit vs. 10 digits in the incorrect solutions.

In summary, in German more errors were produced on the tens (e.g., “twenty” in “four-and-twenty”), whereas errors concerned predominantly the units in French (“four” in twenty-four”). This pattern of results was present for both presentation formats of the additions but was even more prominent in auditory format when task was performed in German and in visual format when task was performed in French.

## Discussion

To provide new insights into the question of bilingual's arithmetic problem solving we tracked arithmetic performances in German-French bilinguals at five different stages of their bilingual development from adolescence to adulthood. Four age-groups of pupils attending secondary school and one group of young adults had to provide oral answers to simple (i.e., addends <10) and complex (i.e., addends >10) addition problems presented once in a visual format (Arabic digits) and once in an auditory format (spoken number words). Moreover, all participants performed experimental tasks both in German and French in two distinct language sessions. Task language had a direct influence on solving complex addition problems, whereas only much weaker language effects were observed when participants retrieved answers for simple additions. From adolescence to adulthood complex additions performance considerably improved in both German and French, with especially noteworthy gains of accuracy in auditory-presented calculations. Yet, for complex additions a substantial language-related advantage for German additions remained in highly proficient adult bilinguals both in accuracy and response times. In contrast, participants tended to retrieve simple additions comparably well in German and French with increasing bilingual proficiency. In addition, the specific number word structures of German and French also significantly impacted bilinguals' arithmetic performance. Due to the base-20 structure of large French two-digit words, calculations with large numbers over 70 were less well-succeeded in French than German. Furthermore, the tendency to make errors involving the second position of the number word led bilingual participants to produce more errors on the units when calculating in French and more decade-related errors in German. Firstly, we will discuss how language globally affected task performance and then separately consider simple and complex addition solving. Secondly, we will debate upon the effect of number word structure on bilinguals' arithmetic skills.

### Effects of calculation complexity

Overall additions were performed faster and with fewer errors in German than in French. This task language effect seemed to persist even in highly proficient adult bilinguals. As German was learned first by all participants it can be considered as their predominant language. In addition, it was also their language of arithmetic acquisition. Our results are consequently in agreement with the fact that (a) relative language predominance is known to promote arithmetic performance in bilinguals (Marsh and Maki, 1976; McClain and Huang, 1982; Frenck-Mestre and Vaid, 1993; Geary et al., 1993) and (b) bilingual adults solve numerical problems more proficiently in the language in which arithmetic was learned (Bernardo, 2001; Salillas and Wicha, 2012). The results also fit with the idea of non-selective language activation in bilinguals. Thus, lower arithmetic performances in French might also—at least partially—be due to less efficient access for French (in general) than for the predominant German (Bialystok, 2009; Kroll et al., 2013, 2014).

Nevertheless, a more nuanced picture emerged when considering separately how performance in simple and complex additions varied between the increasing language proficiency levels. With simple additions (e.g., 4 + 3 = 7) seventh and eighth graders were still marginally less accurate in French than German. But all other participants from grade 10 and upward did not show any accuracy difference between German and French when solving simple additions. If arithmetic acquisition language alone would explain language-related differences in bilinguals' arithmetic performance, then we would have expected an advantage for simple additions in the German session persisting in all age groups. However, here we observed that after 3 years of math-classes in French (i.e., grade 10 and upwards) participants solved simple additions with equal accuracy levels in German and French. This suggests that in addition to the importance the language for arithmetic acquisition, the current language proficiency level modulated the ability to retrieve simple arithmetic facts. Once a certain proficiency level was reached in both of the bilinguals' languages, the initial advantage for solving simple additions in the language in which they had been acquired (i.e., German) no longer applied for accuracy rates, as participants attained ceiling performances for these very simple arithmetic problems. However, even in adults some response time differences between languages remained, though reduced in comparison to other age-groups.

In complex addition (e.g., 54 + 13 = 67), language-related performance differences were more prominent as responses remained slower and less accurate in French than in German in all groups, which is consistent with the idea that complex additions require more processing steps and are therefore more likely to be influenced by language (Beishuizen, 1993). At first sight, raw data analysis indicated that complex addition response times of both language sessions became increasingly similar with age. However, when group differences in variance were eliminated by data standardization it appeared that language-related performance differences in favor of German remained of similar importance across all age groups. Concerning German, even with mathematics taught in French during the entire secondary school years, we observed neither decrease nor stagnation of arithmetic performances in comparison to French. Thus, complex calculation proficiency in the first language (i.e., German) seems to pursue a continuous development independently of the language in which formal math education is taught.

Complex additions were also affected differentially by presentation format of the additions, whereas no substantial difference was observed in simple additions. Participants made always more mistakes with auditory-presented additions. But French still enhanced this effect, with participants making on average 34% (± 0.01% SE) errors when computing auditory-presented complex additions (vs. 22% (± 0.01 SE) errors in German). Over and above this interaction with task language, auditory-presented complex additions were succeeded less well than visually-presented ones. However, the auditory disadvantage gradually reduced with increasing age (even in standardization data). This relative improvement for auditory-presented additions that was specific to complex problems might be due to developmental trends in cognitive and verbal abilities combined with a prolonged exposure to complex addition solving and an increasing math expertise. Indeed, as attested by the ceiling performances observed in simple additions, all participants were perfectly skilled to retrieve arithmetic facts, coherently with the common observation that children usually achieve arithmetic fact retrieval around the age of 8 years onwards (Barrouillet and Fayol, 1998; Butterworth, 2005). Although, participants' performances on complex additions did not reach any ceiling and continued to improve across age-groups in both languages. These observations fit well with the idea that solutions for complex additions cannot be retrieved directly from memory, even in adults (Ashcraft, 1995).

For this type of complex arithmetic computation, factors such as procedural knowledge, planning and working memory are known to play critical roles (Fürst and Hitch, 2000). In auditory presentation format, the additional need to keep the heard addends in working memory may interfere with using the phonological loop in the computation process. Consequently, participants made more errors for auditory presented additions than visually presented additions. This format effect in complex additions was especially pronounced when performing the additions in French, i.e., a language that was relatively less proficient (LeFevre et al., 2001) and/or distinct from the language of arithmetic acquisition. These findings nicely highlight the involvement of language in the numerical processing underlying complex additions. If participants had simply computed the results in their first language (i.e., German) and then translated them to the output language (i.e., French) this would have affected performance similarly in both the visual and the auditory presentation formats. But contrary to this prediction performance specifically dropped when participants computed auditory-presented complex additions in French. This may be due to the fact that arithmetic was learned in German or to globally weaker proficiency level in French. Due to the specific differences between number word structure in French and German languages, the interaction might also (at least partially) result from differences between French and German number naming systems. In the following paragraphs, we will further discuss the latter effects and their relation to arithmetic in German-French bilinguals.

### Effects of number word structure

Languages differ in the way they construct two-digit number words (Campbell and Xue, 2001). This may directly influence bilinguals' addition skills and/or interact with other factors such as bilingual proficiency level and arithmetic acquisition language. Evaluating how arithmetic problem solving is influenced by number word structure in German-French bilinguals is particularly interesting because those both languages encounter two major differences in their number naming systems. Firstly, two-digit number words follow a unit-ten order in German (e.g., “24” = four-and-twenty) but a ten-unit order in French (e.g., “24” = twenty-four). Secondly, the 10 words for the numbers over 70 follow a base-10 structure in German (e.g., “72” = two-and-seventy) but a base-20 structure in French (e.g., “72” = sixty-twelve).

To characterize the effect of number word differences between languages on arithmetic performances, we conducted additional analyses on complex additions. Firstly, we focused on the base-10 vs. base-20 structure of large two-digit number words. Additions involving numbers under and over 70 were analyzed separately, since number words under 70 follow a base-10 structure in both language but number words over 70 follow a base-ten structure in German and a base-20 structure in French (e.g., “72” is pronounced as “sixty-twelve”). Not surprisingly, additions over 70 were solved overall slower than additions under 70 in both languages, confirming the classical problem size effect (Groen and Parkman, 1972). Nevertheless, the response time difference between additions under and over 70 was larger in French than in German. Moreover, in terms of accuracy, participants made more errors for additions over 70 than additions under 70 in French, but not in German where errors rates in additions under and over 70 were similar. Interestingly, these results were observed regardless of additions' presentation formats and participant groups. The latter observation demonstrates that the base-10 vs. base-20 effect is not modulated by bilingual proficiency groups. Nevertheless, it remains to be empirically determined whether specific difficulties for number words also occur in French-German bilinguals with French as first language. These findings confirm the early reports by Ellis and Hennelly (1980) that bilinguals' arithmetic skills are inevitably marked and modulated by the number word structure of the language in which they are currently calculating. In line with the present results, recent behavioral and electrophysiological studies indicate that these language-related characteristics might even impregnate basic number representations (Pixner et al., 2011; Salillas and Carreiras, 2014).

Secondly, we analyzed the type of errors participants made, namely whether more errors were made on the tens or on the units across different languages, presentation formats, and bilingual proficiency groups. As noted above, the number word structure in French and German differs in terms of which digit is pronounced at first in two-digit number words (ten vs. unit). It appeared that participants systematically produced more errors on the ten digit (e.g., “2” in “24”) when calculating in German and more errors on the unit digit (e.g., “4” in “24”) in French. Again, the presentation format and the group of participants did not modify this result. Thus, independent of the calculation language, errors seem to predominantly concern digits holding the second position of the solution number. These findings elegantly show how a language-independent focus on the first segment of number words can lead to qualitatively distinct numerical outcomes within different language contexts. Taken at face value, they imply that making calculation errors while computing prices in the range between 18 and 100 will become more expensive for a German- than for a French-speaking person.

## General considerations

Literature provides divergent conclusions about the level at which bilinguals' different languages are involved in number processing and about the language in which bilinguals actually solve arithmetic problems. Many factors such as age of acquisition of the second language, language of teaching during school years and currently used language seem to determine the use of the language during arithmetic problem solving in bilinguals (Bernardo, 2001; Campbell and Epp, 2004; Salillas and Wicha, 2012). Investigating arithmetic performance in Luxembourgish adolescents and young adults who become highly proficient German-French bilinguals through the school system offered the rare opportunity to study large groups of bilingual participants at different bilingual proficiency levels who are homogeneously composed with respect to the previous factors.

Our findings obtained with German-French bilinguals at five distinct levels of bilingual proficiency extend the current knowledge by confirming that language plays a critical role in the computations underlying complex addition (i.e., operands above “10”) at all bilingual stages. Participants' skills in computing additions in both German and French improved steadily with increasing bilingual proficiency levels from grade 7 to young adulthood. Nevertheless, participants of all age groups solved complex German additions faster and more accurately than French ones. This German advantage remained although mathematics is taught in French during the entire secondary school years. It is probably due to the fact that German is participants' first school language and their arithmetic acquisition language and that complex additions are not automatized enough to be free of any language help along the solving process. In contrast, simple addition facts (i.e., operands below “10”) were accessed more directly and similarly in both languages, especially at later stages of second language acquisition. Indeed accuracy levels for simple additions were similar in French and German from grade 10 upwards, while their response times got closer. Thus, highly proficient bilinguals tend to be able to retrieve addition facts similarly in both languages suggesting that bilinguals' arithmetic fact retrieval may become either independent from the verbal code or automatized enough in different languages' verbal codes to lead to similar performances (Campbell and Epp, 2004).

The second part of our study explored the role of number word structure in bilinguals' arithmetic performance. German-French bilinguals indeed speak two languages that are characterized by inverted ten-unit structures of two-digit number words (unit-ten vs. ten-unit number words) and with different constructions of tens over 70 (base-10 vs. base-20). Consequently the full effect of number word structure on arithmetic computation could be highlighted optimally in this type of bilingual population. When additions were computed in French, specific response-delays and error-increases were observed for calculations involving number words over 70. Moreover, results from error analyses showed that participants of all age groups always committed more errors related to the digit that occurred in second position in the number word, i.e., tens in German and units in French. Taken together, both differences in German vs. French number word structures (two-digit words with base 10 vs. 20 and direct vs. inverted digit order) seemed to play a role in arithmetic processing at all bilingual proficiency stages.

In conclusion, the present study demonstrates that both (a) language proficiency levels and (b) number word structure affect addition solving performances in bilinguals. This leads to the conclusion that arithmetic significantly relies on language processes, especially in complex computations. Further studies will be needed to generalize the present findings to other number processing tasks (e.g., magnitude comparison), other arithmetic operations (e.g., subtraction, multiplication,) and other tasks with number words (e.g., math word problems).

### Conflict of interest statement

The authors declare that the research was conducted in the absence of any commercial or financial relationships that could be construed as a potential conflict of interest.

## Footnotes

^1^Direct differences of RTs between formats of presentation can only be interpreted as modality-related measurement differences because RT recording started at the end of stimulus presentation in auditory format and at the beginning of stimulus presentation in visual format.
