# Supplementary material for: The relation between language and arithmetic in bilinguals: insights from different stages of language acquisition
Source: Front Psychol. 2015 Mar 13;6:265. doi: 10.3389/fpsyg.2015.00265 (PMC4357777; doi:10.3389/fpsyg.2015.00265)
Supplement: Supplementary file 1 [file Presentation1.PDF]

The relation between language and arithmetic in bilinguals: insights from different stages of language acquisition

Amandine, Van Rinsveld*[a], Martin, Brunner[b], Karin, Landerl[c], Christine, Schiltz[a1], Sonja, Ugen[ad1].

[a] Institute of Cognitive Science and Assessment, Education, Culture, Cognition and Society, University of Luxembourg, Walferdange, Luxembourg.
[b] Free University of Berlin and Berlin-Brandenburg Institute for School Quality, Berlin, Germany
[c] Department of Psychology, University of Graz, Graz, Austria
[d] Luxembourg Center for Educational Testing, University of Luxembourg, Luxembourg, Luxembourg.

[1] These authors equally contributed to this work.

*** Correspondence:** Amandine Van Rinsveld, ECCS research unit - University of Luxembourg, Route de Diekirch, L-7201 Walferdange, Luxembourg, email: amandine.vanrinsveld@uni.lu

Keywords: Numbers; Language learning; Bilingualism; Arithmetic; Addition

Supplementary Material

Annex 1: Additional analyses of section A. - Standardization per age-group

We calculated Z-scores within each age-group by using the general mean and standard deviation of the group but always separately for simple and complex additions. Below we report the results of these additional analyses on standardized data, focusing only on the new outcomes concerning the interactions with the factor "age-groups" since such interactions might have been masked by the heterogeneity of the age-groups in analyses on raw data. Interactions with the age-group factor remaining significant after standardization of the data reveal differences in the effects between groups that are not due to heterogeneity differences between age-groups.

For simple additions, interaction between task-language and age-group on CRs was marginally significant, F (1, 185) = 2.242; p = .066; η^2 = .046. We decomposed the interaction by running separate ANOVAs in each group of participants and results showed that the task-language effect on CRs was again marginally significant for 7th, F (1, 34) = 3.804; p = .059; η^2 = .101, but also for 8th graders, F (1, 31) = 3.317; p = .078; η^2 = .094, and not for the three older groups where participants were as accurate in French as in German, all Fs<1 and ps>.1 . Taken together, the result obtained with standardized and raw data consequently confirm that pupils in the lower secondary grades make more errors when computing simple additions in French than in German.

For complex additions, the task-language effect on RTs was no longer modulated by the group F (1, 185) = 0.454; p = .770; η^2 = .010, showing that, after standardization of the data, all age-groups participants were similarly slower to solve additions in French than in German (see Figure 6; and Figure 2c for raw data results). Nevertheless, the effect of presentation format on the CRs (participants were less accurate with auditory than with visually presented addition) was still modulated by age-group, F (1, 185) = 3.701; p = .006; η^2 = .074 (Figure 7, and see Table 3. for raw data results). We applied the same method as with simple additions to decompose this interaction and

results showed that the difference in CRs between auditory and visual presentation formats decreased with age-group (7th graders: F (1, 34) = 76.478; $p < .001$; $\eta^2 = .692$, 8th graders: F (1, 31) = 31.828; $p < .001$; $\eta^2 = .499$, 10th graders: F (1, 33) = 51.986; $p < .001$; $\eta^2 = .621$, 11th graders: F (1, 39) = 26.211; $p < .001$; $\eta^2 = .396$, adults: F (1, 47) = 31.087; $p < .001$; $\eta^2 = .398$). Thus, the standardized data further highlight the observation that response accuracy in the two presentations formats (verbal vs. visual) became increasingly similar with increasing age.

Annex 2. Additional analyses of section B.1. - Standardization per language

In order to exclude possible confounds of bilinguals' language proficiency on problem size effects, we standardized the data of complex additions per language. Therefore, we used general means and standard deviations of each language of the task for all participants, presentation formats and problem sizes. Below we report the results of these additional standardized analyses, focusing only on the persistence of the interaction between problem size and language, since such interactions might previously have been an artifact of the lower language proficiency in L2 vs. L1 in analyses on raw data. Results showed that interaction between language and problem size was still significant after standardization per language in both RTs, F (1, 173) = 7.205; $p = .008$; $\eta^2 = .040$, and CRs, F (1, 182) = 33.304; $p < .001$; $\eta^2 = .155$. Pairwise comparisons showed that problem size effect was larger in French (RTs: F (1, 173) = 91.565; $p < .001$; $\eta^2 = .346$, & CRs: F (1, 182) = 97.822; $p < .001$; $\eta^2 = .350$) than in German (RTs: F (1, 173) = 64.836; $p < .001$; $\eta^2 = .273$, & CRs: F (1, 182) = 6.037; $p = .015$; $\eta^2 = .032$). Even after standardization per language, for problems involving numbers over 70, French additions were still solved marginally slower than German additions (F (1, 173) = 3.190; $p = .076$; $\eta^2 = .018$), whereas no difference was observed for problems under 70 (F (1, 173) = 1.377; $p = .242$; $\eta^2 = .008$). Thus, interactions of language and problem size remained significant after standardization of the data per language.

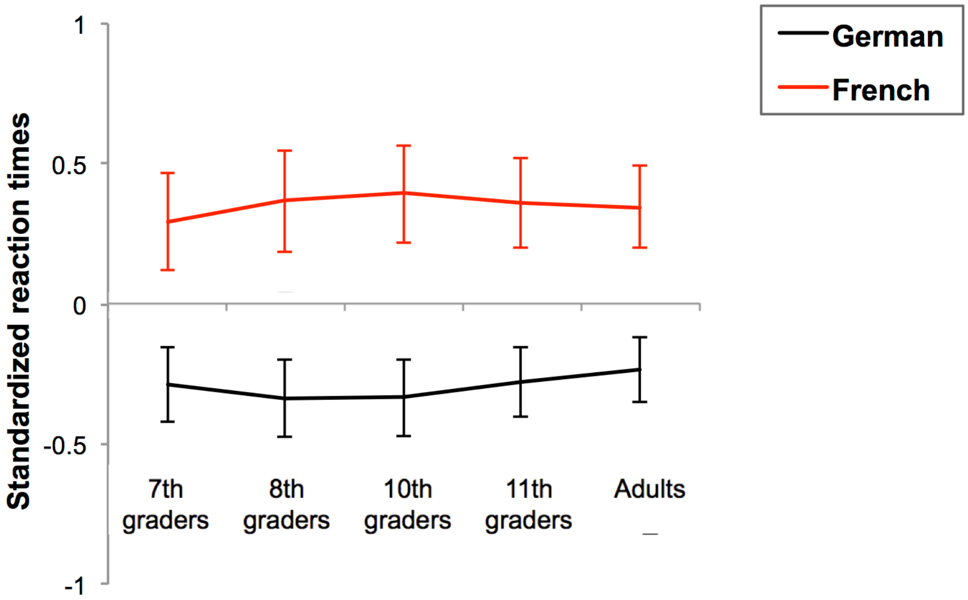

Figure 6. Standardized mean reaction times with standard errors for complex additions in each task language (black line for German and red line for French) as a function of age-group.

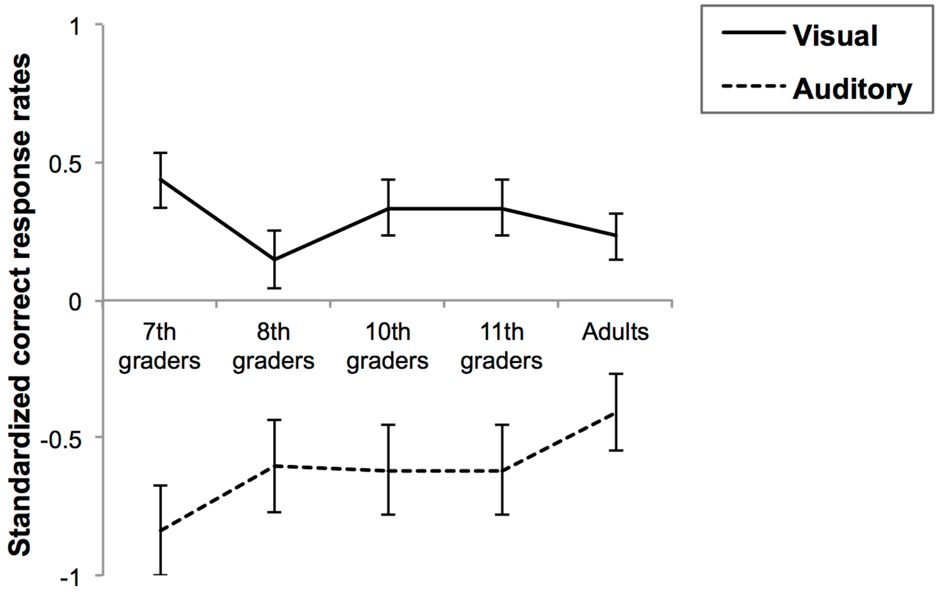

Figure 7. Standardized mean correct response rates with standard errors for complex additions in in each presentation format (solid line for visual and dashed line for auditory) as a function of age-group.
